# Case Report: Sequential use of tezepelumab and mepolizumab for eosinophilic otitis media in EGPA: a steroid-sparing strategy

**DOI:** 10.3389/falgy.2025.1661047

**Published:** 2025-10-23

**Authors:** Osamu Matsuno, Masahiro Kawamoto, Tansri Wibowo, Yutaka Ishida, Atsuhsi Ogata

**Affiliations:** 1 Department of Allergic and Rheumatoid Disease, Osaka Habikino Medical Center, Habikino, Osaka, Japan; 2 Kawamoto Clinic, Nara, Japan

**Keywords:** eosinophilic granulomatosis with polyangiitis (EGPA), mepolizumab, tezepelumab, eosinophilic otitis media, steroid-sparing, biologics, TSLP

## Abstract

Even in eosinophilic granulomatosis with polyangiitis (EGPA), not all manifestations of eosinophilic inflammation respond equally to anti–interleukin-5 (IL-5) therapy. We report a case of steroid-refractory eosinophilic otitis media (EOM) in a patient with EGPA, where systemic symptoms such as asthma and chronic rhinosinusitis initially responded to high-dose mepolizumab, but relapsed upon corticosteroid tapering. Introduction of tezepelumab led to marked improvement in EOM and upper and lower airway symptoms. To sustain remission, we employed a bi-monthly alternating regimen of tezepelumab and mepolizumab, achieving long-term control without dual biologic use or systemic corticosteroid escalation. This case highlights a personalized and steroid-sparing strategy for managing complex type 2 inflammation in EGPA.

## Introduction

Not all eosinophilic inflammation is created equal. In eosinophilic granulomatosis with polyangiitis (EGPA), upper and lower airway manifestations—including chronic rhinosinusitis and eosinophilic otitis media (EOM)—can persist or relapse despite high-dose anti–IL-5 therapy (1). This suggests involvement of upstream drivers such as thymic stromal lymphopoietin (TSLP) or IL-4/IL-13 pathways.

Biologics have also been reported effective in EOM in non-EGPA contexts, including cases treated with dupilumab (2), mepolizumab (3, 4), and various biologics in a small series (5). Although rare, reports of EGPA-related otologic manifestations exist, such as EOM controlled with mepolizumab (6) and a case requiring combination benralizumab and dupilumab to stabilize otologic and sinonasal disease (1). These observations illustrate both the potential and the complexity of biologic use in EOM and EGPA. Here, we present a case of steroid-refractory EGPA-associated EOM successfully managed with a staggered regimen alternating tezepelumab and mepolizumab, without simultaneous dual biologic exposure. This approach achieved sustained, steroid-sparing remission of EOM while maintaining control of sinonasal and lower airway symptoms. This approach may offer a practical and more accessible strategy for managing complex, multi-compartment type 2 inflammation in EGPA, particularly in settings where the cost or availability of concurrent biologics poses a challenge. Although no formal cost analysis was conducted, alternating use of two biologics on a monthly basis, rather than concurrent administration, intuitively reduces drug-related expenses by approximately half.

## Case report

A 61-year-old woman with severe asthma and chronic rhinosinusitis with nasal polyps (CRSwNP) was referred for progressively worsening EOM, characterized by conductive hearing loss, persistent otorrhea, and middle ear granulation. Her asthma had been diagnosed at age 42, and CRSwNP at age 44. According to the 2022 American College of Rheumatology/European Alliance of Associations for Rheumatology (ACR/EULAR) classification criteria for EGPA (7), the patient met the following criteria: obstructive airway disease (+3), nasal polyps (+3), mononeuritis multiplex (+1), and blood eosinophilia ≥1,000/μl (+5). C-ANCA was negative, and hematuria was absent, neither of which reduced the total score. The cumulative score was 12 (≥6), thereby supporting the classification of EGPA. The patient was MPO-ANCA (P-ANCA) positive (36.8 IU/ml); however, this is not a weighted item in the scoring system.

Initial treatment included steroid pulse therapy followed by high-dose oral corticosteroids (prednisolone 40 mg/day) and oral cyclophosphamide (50 mg/day), which led to improvement of pulmonary and cutaneous manifestations. However, corticosteroids remained necessary and neuropathic symptoms persisted, so high-dose intravenous immunoglobulin (20 g/day for 5 days) was subsequently administered twice. After tapering corticosteroids to 10 mg/day, the patient's EOM worsened at age 53, presenting with viscous otorrhea containing numerous eosinophils. She was treated with tympanic membrane incision, betamethasone ear drops, and triamcinolone ear bath, with temporary improvement.

Mepolizumab (300 mg every 4 weeks) was initiated at age 56 due to worsening asthma and EOM symptoms, resulting in initial improvement and allowing steroid tapering to 5 mg/day. Blood eosinophil counts, which had risen to 300/μl at the time of asthma and EOM recurrence, declined to 60/μl after 3 months of mepolizumab therapy during the initial treatment phase. However, despite this hematologic response, the patient's EOM and asthma symptoms gradually worsened. At age 61, her disease had become refractory, and prednisolone had to be increased to 15 mg/day without sufficient benefit, prompting consideration of alternative therapy.

**Figure 1:**
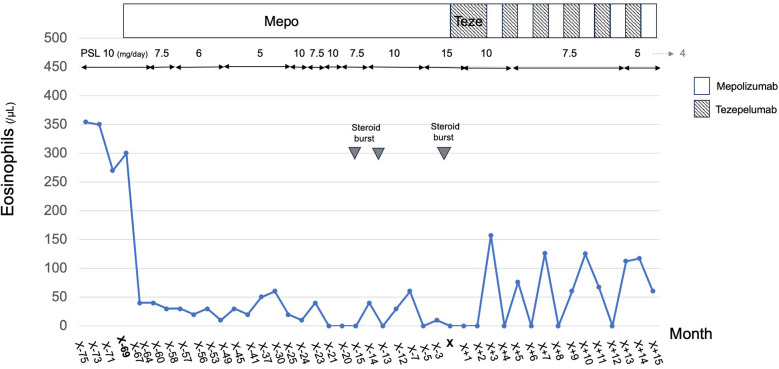
Swimlane-style timeline illustrating the clinical course and treatment transitions in a patient with eosinophilic granulomatosis with polyangiitis (EGPA) and eosinophilic otitis media (EOM). The figure displays the temporal relationship between clinical events, oral prednisolone (PSL) dosing, peripheral eosinophil counts, and the administration of biologics (mepolizumab and tezepelumab). The timeline begins with the initiation of biologic therapy and highlights the lack of sustained control with mepolizumab monotherapy, followed by the introduction of tezepelumab and the subsequent alternation of the two agents at four-week intervals. Steroid bursts and clinical relapses are also indicated, providing a visual summary of disease activity and therapeutic response.

**Figure 2:**
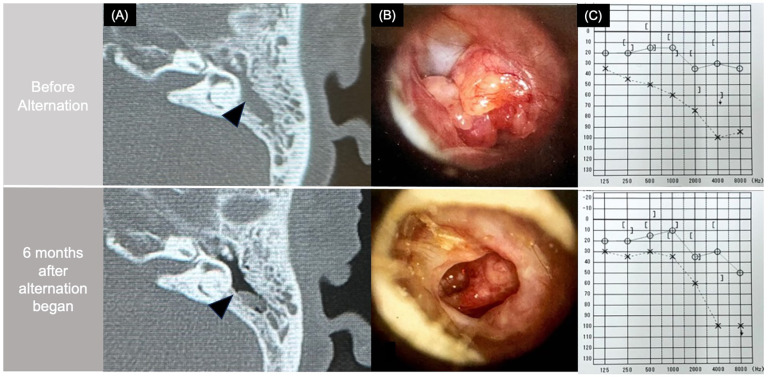
Computed tomography (CT), otoscopic, and audiometric changes before and after alternating therapy. **(A)** CT scan showing middle ear opacification before therapy (top) and resolution after therapy (bottom). **(B)** Otoscopy demonstrating reduction of granulation tissue after treatment. **(C)** Audiometry showing hearing improvement following alternating therapy.

Given the insufficient clinical response, subcutaneous tezepelumab, a human monoclonal antibody targeting TSLP, was initiated at age 60 at a dose of 100 mg every 4 weeks. This alternating strategy was selected based on the pharmacokinetics and approved dosing intervals of both biologics, which are typically administered every 4 weeks. We believe this approach maintains therapeutic drug levels while minimizing potential additive immunosuppressive effects. At that time, the patient was receiving high-dose triple inhaler therapy (ICS/LABA/LAMA, Enerzair®) in combination with oral theophylline (Theodur® 400 mg/day). This upstream TSLP inhibitor led to improvement in asthma symptoms, nasal obstruction, FeNO levels (declining from 46 ppb to 19 ppb), as well as in EOM manifestations (8, 9). After three consecutive monthly doses of tezepelumab, the administration of tezepelumab and mepolizumab was alternated monthly, with each agent given every other month (Figure 1). Within three months of initiating this staggered regimen, otoscopic findings showed marked improvement. Soft tissue opacities in the tympanic cavity and mastoid antrum diminished, leading to improved aeration and clearer visualization of the ossicular chain on CT. Before alternating therapy, the patient had severe sensorineural hearing loss in the left ear, with a three-frequency pure-tone average (air conduction) of 61.7 dB and a corresponding bone conduction average of 66.7 dB. After 6 months of alternating therapy, the air conduction threshold improved to 41.7 dB, and bone conduction improved to 51.7 dB, indicating both conductive and sensorineural improvement (Figure 2). These improvements support both anatomic and functional resolution of EOM. Because mepolizumab alone did not control otologic manifestations and asthma, and simultaneous dual biologic therapy was avoided, a monthly alternating regimen with tezepelumab was adopted to maintain remission while allowing corticosteroid tapering.

Systemic vasculitic manifestations (renal, cutaneous, neurologic, or pulmonary hemorrhage) did not recur, and disease activity remained confined to EOM and asthma. Therefore, BVAS, VDI, and SNOT-22 scores were not collected. Instead, the patient was monitored using serial FeNO measurements, ACT scores, chest CT imaging, otoscopic findings, and audiometry, which provided objective and disease-specific outcome measures. Importantly, this therapeutic sequence was well-tolerated, and the patient experienced no adverse events related to either biologic throughout the treatment course. Her Asthma Control Test (ACT) score improved from 18 to 23 following the switch. Serial FeNO and peripheral eosinophil counts remained suppressed during the alternating regimen, suggesting a sustained anti-inflammatory effect through both upstream and downstream targeting. At the time of tezepelumab initiation, MPO-ANCA had decreased to <0.2 IU/mL and remained below this level throughout follow-up, indicating no systemic vasculitic relapse during the alternating biologic regimen.

Audiometry revealed improvement in air conduction threshold in the left ear, consistent with the patient's subjective improvement in hearing. Her sinonasal symptoms, including postnasal drip and nasal obstruction, also diminished significantly over time**.** As disease control stabilized under the alternating regimen, oral prednisolone was successfully tapered from 15 mg/day to 5 mg/day over 12 months, without recurrence of otologic or respiratory symptoms. Imaging follow-up at 6 months showed sustained resolution of middle ear opacification without need for additional surgical intervention. Middle ear fluid collected at baseline demonstrated eosinophilic predominance on cytological smear, confirming the diagnosis of EOM. Repeat smear after treatment showed resolution of inflammatory infiltrate.

From a mechanistic standpoint, this biologic sequence is rational. Tezepelumab acts upstream in the type 2 inflammatory cascade by blocking TSLP, broadly suppressing epithelial cytokine signaling without provoking eosinophilia (6, 8, 10). In contrast, mepolizumab targets IL-5, directly inhibiting eosinophilic proliferation and survival (3, 4). Dupilumab, though effective for CRSwNP and ABPA, has been associated with paradoxical eosinophilia and EGPA flares when used as monotherapy (11, 12).

These findings support selection of tezepelumab as a first step in biologic sequencing, particularly in patients with prominent epithelial inflammation. However, the use of tezepelumab in EGPA remains investigational, and its optimal dosing and efficacy in preventing systemic vasculitic flares are currently unknown (13). Our cycling approach, in which tezepelumab and mepolizumab were administered alternately every month, achieved durable control of both systemic and local disease manifestations without concurrent administration, offering a clinically effective and economically sustainable alternative (14).

Our case aligns with earlier Japanese reports of alternating benralizumab and dupilumab in patients with severe eosinophilic asthma and EOM (14). However, to our knowledge, this is the first report applying cycling therapy to EGPA-associated otologic disease with radiologic and audiometric confirmation. Notably, the Japanese diagnostic criteria for EOM exclude EGPA, yet our case exhibited all core features of EOM, including eosinophil-rich otorrhea, steroid-refractory middle ear inflammation, comorbid asthma, and CRS.

Furthermore, a genome-wide association study (GWAS) identified TSLP as a susceptibility gene in EGPA, lending pathophysiologic credibility to TSLP inhibition (9). This supports the rationale for biologic sequencing based on disease phenotype—initially epithelial-driven, followed by persistent eosinophilic activity—to optimize treatment response.

The phased nature of this strategy also offers potential advantages for pharmacovigilance and stepwise evaluation of therapeutic response. While alternating therapy was associated with clinical benefit in our patient, the efficacy and safety of such an approach in EGPA remain uncertain and should be validated in future studies. As cost constraints and drug availability vary across countries and healthcare systems, such flexible, personalized strategies may be particularly valuable in achieving long-term disease control without overreliance on high-dose corticosteroids or prolonged dual biologic use (15).

This case illustrates the potential for personalized, phased biologic strategies in managing complex type 2 inflammatory diseases. While no formal pharmacoeconomic analysis was performed, the alternating regimen may represent a cost-conscious and steroid-sparing option. Additionally, since tezepelumab is currently used off-label for EGPA, this approach may have implications for real-world accessibility and reimbursement, particularly in healthcare systems with restricted biologic formularies.

## Perspective

The patient expressed satisfaction with symptom improvement and reduced reliance on corticosteroids.

## Data Availability

The datasets presented in this article are not readily available because this is a single-patient case report. No datasets were generated or analyzed. All relevant clinical information is included in the manuscript. Requests to access the datasets should be directed to Osamu Matsuno, matsunoopra.opho.jp.
